# Panic Attacks and Cervical Pain: Outcomes of Traditional and Anti-Oxydative Therapy

**DOI:** 10.1192/j.eurpsy.2023.453

**Published:** 2023-07-19

**Authors:** D. Labunskiy, N. Kurgaev, N. Kolmykova, V. Podsevatkin, S. Kiryukhina

**Affiliations:** Ogarev Mordovia State University, Saransk, Russian Federation

## Abstract

**Introduction:**

Panic attacks(PA) or episodic paroxysmal anxiety are attacks of severe anxiety (panic) or fear (most often - fear of death, less often - fear of losing consciousness, loss of control, helplessness or fear of “going crazy”), accompanied by a rapid heartbeat and a feeling of “suffocation” , “lack of air.” Sometimes there are additional symptoms such as increased blood pressure, a feeling of “internal trembling”, trembling in the limbs, a feeling of “hot flashes” of heat or cold, numbness of the extremities, increased sweating, a feeling of “unstability” or dizziness, nausea, derealization or depersonalization.

**Objectives:**

It was revealed that many patients with cervical and spinal pain suffer from PA. Psychotherapy and traditional psychopharmacology treatment often not effective. The goal of our study was analysis of radical anti-oxidative therapy for the PA patients in addition to traditional to traditional psychoparmacology and psychotherapy techniques.

**Methods:**

12 patients with cervical and 31 with lumbar pain experienced panic attacks during outbreaks of panic attacks. 6 cervical pain patient and 17 patients with lumbar pain were treated by antipsychotic medications also by cognitive behavioral therapy, hypno-suggestive therapy and autogenic training. Other patients also receive hyperbaric oxidation therapy (HBO) in addition to psychopharmacology and psychotherapy.

**Results:**

It was revealed that patients with PA comorbid with vertebral pathology had much better effects in terms of evading of psychopathologic outcomes. PA became much more rarely and finally disappeared at all. The anti-oxidative treatment was also very beneficial for neurologic symptoms cause by vertebral pain.

**Conclusions:**

Anti-oxidative therapy showed very marked effect. In this regard, new anti-oxidative treatment seems to be promisable for management of such conditions.

**Disclosure of Interest:**

None Declared

